# Aldehyde Dehydrogenases Function in the Homeostasis of Pyridine Nucleotides in *Arabidopsis thaliana*

**DOI:** 10.1038/s41598-018-21202-6

**Published:** 2018-02-13

**Authors:** Tagnon D. Missihoun, Simeon O. Kotchoni, Dorothea Bartels

**Affiliations:** 1 Institute of Molecular Physiology and Biotechnology of Plants (IMBIO), University of Bonn, 53115 Bonn, Germany; 2 Department of Biology, Rutgers University, 315 Penn St., Camden, NJ 08102 USA; 3 Center for Computational and Integrative Biology, Rutgers University, 315 Penn St., Camden, NJ 08102 USA; 4 Present Address: Department of Microbiology and Plant Pathology, University of California Riverside, Riverside, CA 92521 USA

## Abstract

Aldehyde dehydrogenase enzymes (ALDHs) catalyze the oxidation of aliphatic and aromatic aldehydes to their corresponding carboxylic acids using NAD^+^ or NADP^+^ as cofactors and generating NADH or NADPH. Previous studies mainly focused on the ALDH role in detoxifying toxic aldehydes but their effect on the cellular NAD(P)H contents has so far been overlooked. Here, we investigated whether the ALDHs influence the cellular redox homeostasis. We used a double T-DNA insertion mutant that is defective in representative members of *Arabidopsis thaliana* ALDH families 3 (ALDH3I1) and 7 (ALDH7B4), and we examined the pyridine nucleotide pools, glutathione content, and the photosynthetic capacity of the *aldh* mutants in comparison with the wild type. The loss of function of ALDH3I1 and ALDH7B4 led to a decrease of NAD(P)H, NAD(P)H/NAD(P) ratio, and an alteration of the glutathione pools. The *aldh* double mutant had higher glucose-6-phosphate dehydrogenase activity than the wild type, indicating a high demand for reduced pyridine nucleotides. Moreover, the mutant had a reduced quantum yield of photosystem II and photosynthetic capacity at relatively high light intensities compared to the wild type. Altogether, our data revealed a role of ALDHs as major contributors to the homeostasis of pyridine nucleotides in plants.

## Introduction

Aldehyde dehydrogenase enzymes (ALDHs) represent an evolutionary conserved protein superfamily present in nearly all organisms^1,2^, and up to now, 16 ALDH genes have been identified in *Arabidopsis thaliana*, and are classified in 10 protein families^2^. Several Arabidopsis ALDHs from family 3, 7 and 10 have been shown to play a role in plant stress responses and development^3–5^. For example, the overexpression of ALDH3I1 and ALDH7B4 conferred abiotic stress tolerance to Arabidopsis and tobacco plants^6–8^, and the reduced epidermal fluorescence1 (*ref1*) phenotype, which resulted from a mutation in the Arabidopsis *ALDH2C4* gene (*At3g24503*), affected the synthesis of ferulic acid and sinapic acid in the phenylpropanoid pathway^9,10^. ALDH2B4, a member of the protein family 2, was shown to be involved in the pyruvate dehydrogenase bypass^11^. These examples demonstrate a role for some plant ALDHs in growth, development and response to environmental stresses (reviewed in ref.^4^). However, the mere transformation of the aldehyde into carboxylic acid often fails to explain how some *ALDH* genes are involved in complex phenotypes such as the nuclear restoration of cytoplasmic male sterility^12,13^, submergence tolerance^14^, seed maturation^15,16^, and cell proliferation^17^. Although the ALDHs use NAD^+^ or NADP^+^ as cofactors and generate NADH or NADPH, studies on both plant and animal ALDHs have only focused on their role in detoxifying toxic aldehydes, and have so far overlooked the potential contribution of the ALDH activity to the cellular NAD(P)H pools and NAD(P)H/NA(D)P ratio. In this study, we examined this hypothesis and found that the NAD(P)H pools were altered in the Arabidopsis *aldh* mutants particularly under conditions for high demand of reducing power.

## Results

### The disruption of *ALDH3I1* and *ALDH7B4* decreased the cellular NAD(P)H contents and altered the NAD(P)H/NAD(P) ratio

**Figure 1:**
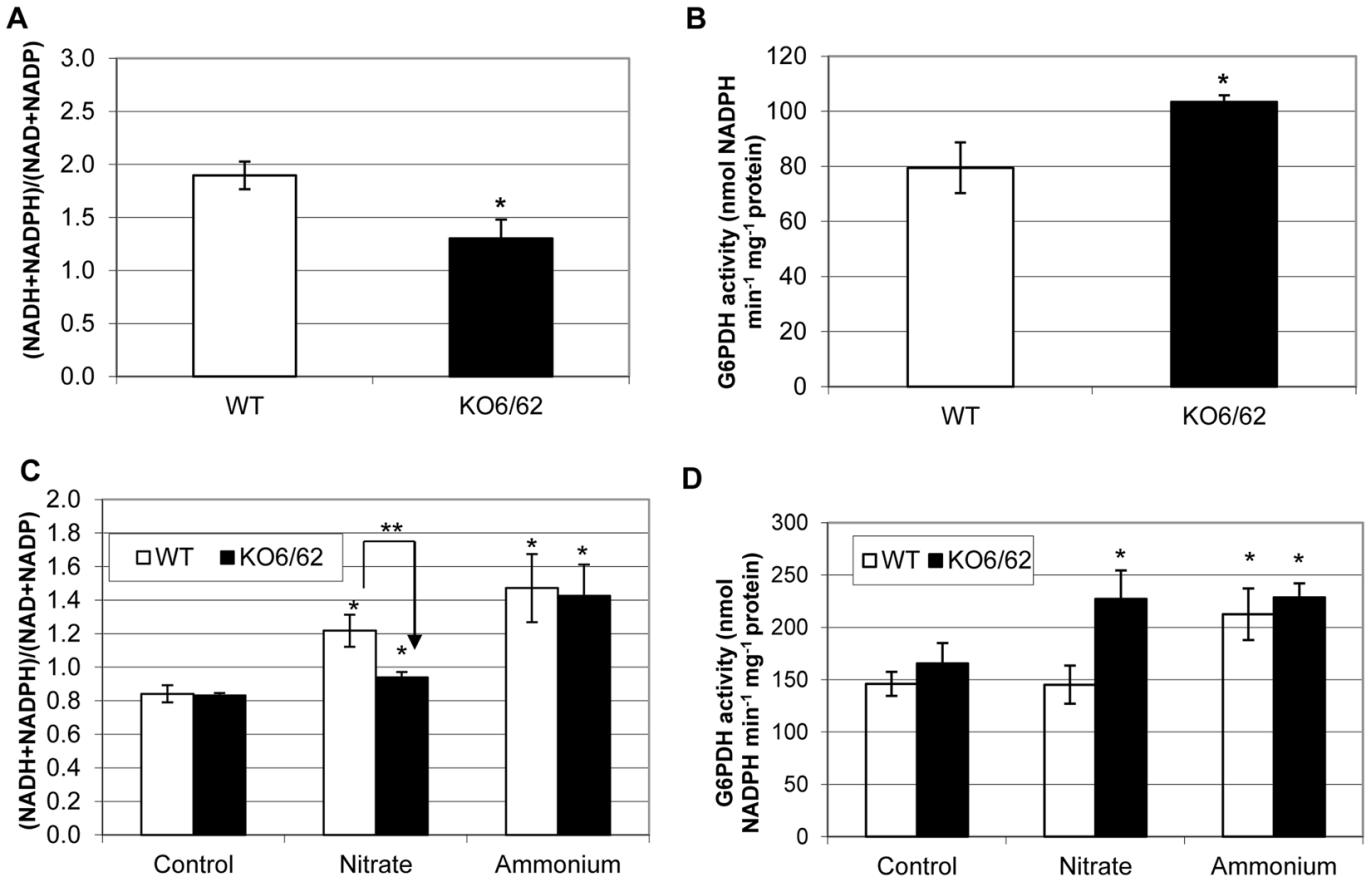
Levels of pyridine nucleotides and glucose-6-phosphate dehydrogenase (G6PDH) activity in WT and *aldh* mutants. (**A**) Ratios of total reduced to oxidised pyridine nucleotides in 6-week-old plants. (**B**) Total G6PDH activity from 6-week-old plants. Data in A and B represent mean values ± SE (n = 9 plants). (**C**) Ratios of total reduced to oxidised pyridine nucleotides in 2-week-old seedlings. (**D**) Total G6PDH activity from 2-week-old seedlings extracts. Data in (**C** and **D**) represent mean values ± SE (n = 4 pools of seedlings). For the measurements, seedlings were grown on half-MS agar plates for 7 days then transferred on fresh plates supplemented with 0 mM (control) or 30 mM of either potassium nitrate (shown as Nitrate) or ammonium chloride (shown as Ammonium). Seedlings were harvested after 7 days of additional growth. Single asterisks in (**A** and **B**) show significant differences between control and treatments whereas the double asterisk in (**C**) indicates significant differences between the accumulation of reduced pyridine nucleotides in WT and *KO6/62* on nitrate (*P* < 0.05; Student’s *t* test).

To investigate whether the ALDHs influence the cellular redox, we examined their contribution to the pyridine nucleotide NAD(P) and NAD(P)H pools by using a double T-DNA insertion mutant (*KO6/62*) of *A. thaliana* that is defective in the *ALDH7B4* and *ALDH3I1* genes. ALDH3I1 and ALDH7B4 were previously shown to be localized in the chloroplast and the cytosol of *Arabidopsis thaliana* cells, respectively, and their expression level increases in response to abiotic stresses^4,18^. Stress-triggered induction of ALDH7B4 and ALDH3I1 was impaired in the single T-DNA insertion lines *KO6* and *KO62*, respectively, and both *KO6* and *KO62* plants accumulated higher levels of reactive oxygen species (ROS) and malondialdehyde (MDA) than the wild type^7^. In this study, we verified that the double mutant *KO6/62* does not express the *ALDH3I1* and *ALDH7B4* genes. Although a transcript was still amplifiable from the *KO6* mutant by using primers specific for *ALDH7B4*, the ALDH7B4 protein level in this line was drastically reduced, as shown for seed protein extracts (Figure S1). Next, we measured the levels of the oxidized and reduced forms of the pyridine nucleotides NAD, NADP, NADH, and NADPH in leaves of six-week-old plants (Table 1). The levels of the reduced pyridine nucleotides were lower in all *aldh* mutants than the wild type. Compared to the wild type, NADH and NADPH were reduced by 27% and 79% in the double mutant *KO6/62*, respectively. In comparison, there were above 50% increase of NAD, the preferred substrate for the plant ALDHs over NADP, in the single *aldh* mutant *KO6* and *KO62*, and 27% increase in double mutant *KO6/62* compared to WT. In contrast to *KO6* that is defective in the cytosolic ALDH7B4, the WT and the mutants *KO62* and *KO6*/62 showed similar levels of NADP. Moreover, the NAD(P)H/NAD(P) ratio significantly decreased by 32% (from 1.9 to 1.3) in the double mutant *KO6/62* compared to WT (Fig. 1A), mainly as a result of a low concentration of the NAD(P)H pools in the ALDH single and double mutants compared to WT.

**Table 1:** Levels of pyridine nucleotides in WT and *aldh* mutants.

	WT	*KO6*	*KO62*	*KO6/62*
NADH	30.55 ± 1.40	**23.73** ± 2.15	**22.67** ± 1.27	**22.43** ± 2.08
NADPH	2.27 ± 1.31	1.11 ± 0.64	**0.45** ± 0.26	**0.47** ± 0.27
NAD	13.11 ± 1.50	**19.55** ± 1.34	24.55 ± 4.81	16.73 ± 1.94
NADP	9.79 ± 0.78	**4.44** ± 1.29	9.79 ± 1.28	9.48 ± 1.10

Values are in nmol g^−1^ FW. Data represent mean values ± SE (n = 9 plants). Numbers in bold show significant differences from the wild type (WT) (*P* < 0.05; Student’s *t* test).

Several studies reported about the sensitivity of the plant glucose-6-phosphate dehydrogenases (G6PDH) to the intracellular level of reduced pyridine nucleotides NADH and NADPH, which manifests by an increase of the G6PDH activity in response to a low cellular level of reduced pyridine nucleotides^19–21^. To verify the alteration of NAD(P)H contents found in the *aldh* mutant, we then measured the total G6PDH activity in the mutant and WT leaf protein extracts. We found that the G6PDH activity was about 20% higher in the double mutants *KO6/62* mutants than in WT (Fig. 1B), confirming the well-known positive regulation of the G6PDH enzyme by low NAPH/NADP ratio^22^. In another experiment, we leveraged the fact that nitrate assimilation by the plant requires substantial amounts of NAD(P)H to further verify the alteration of NAD(P)H contents in the *aldh* mutant. We thus analyzed the pools of NAD(P) and NAD(P)H in wild-type and *KO6/62* seedlings grown on a medium supplemented with nitrate. The rationale was that growing the seeds on a medium supplemented with nitrate would increase the demand of NAD(P)H, and allow any defect in the NAD(P)H pools in the seedlings to become visible. In agreement with this, the NAD(P)H/NAD(P) ratio increased in the WT and in the *KO6/62* mutant when grown on agar plate supplemented with nitrate or ammonium. However, the increase was 18% less in the *aldh* double mutant *KO6/62* (from 0.83 to 0.94–13%) than in WT (from 1.22 to 0.84–31%) in medium supplemented with nitrate (Fig. 1C), which shows that the supply or the consumption of NAD(P)H are altered in the *aldh* mutant. No difference was seen between the *aldh* mutant and WT when they were grown in a medium supplemented with ammonium, suggesting that the supply of NAD(P)H by the ALDH could be more relevant to nitrate assimilation than ammonium assimilation. As in the adult plants, we measured the G6PDH activity in the seedlings grown on the medium supplemented with nitrate. The G6PDH activity was 39% higher in the mutant than in WT, indicating a high demand for NAD(P)H in the *aldh* double mutant compared to WT (Fig. 1D). Similarly to NAD(P)/NAD(P), there were no difference in the G6PDH activity between the *aldh* mutant and WT when grown in a medium supplemented with ammonium. These results, altogether, indicate an alteration of NAD(P)H pool in the *aldh* mutant compared to WT. Nevertheless, the change in the NAD(P)H content was only observable in the leaves of adult mutant plants and not in the seedlings under normal growth conditions, as it can be seen by comparing Fig. 1A and B with the control condition shown in Fig. 1C and D, respectively.

### Disruption of ALDH3I1 and ALDH7B4 affects Glutathione Metabolism

**Figure 2:**
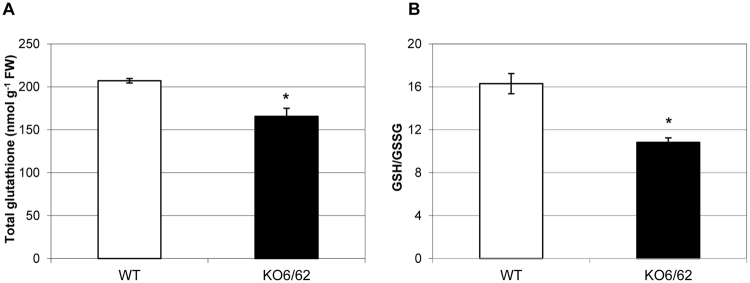
Glutathione pools in WT and *KO6/62* plants. (**A**) Total glutathione denotes reduced plus oxidised nanomole (nmol) glutathione per gram leaf fresh weight (FW). (**B**) GSH/GSSG denotes the ratio of reduced to oxidised glutathione pools. Shown are the mean values ± SE (n = 4 pools of leaves each from 3 plants). Asterisks indicate significant differences between WT and *KO6/62* plants (*P* < 0.05; Student’s *t* test).

We hypothesized that the low level of reduced pyridine nucleotides in the *ALDH* double mutant might also affect the redox state of the glutathione pools given that the turnover of antioxidant pools such as glutathione and ascorbate depends on NAD(P)H availability^23,24^. To verify this hypothesis the glutathione pools were compared in WT and *KO6/62* plants. The levels of the total glutathione (reduced + oxidized) and of the reduced to oxidized glutathione ratio (GSH/GSSG) were reduced by 20% and 33% in *KO6/62* compared to WT, respectively (Fig. 2). This would reflect a change in the cellular redox homeostasis following the inactivation of the *ALDH3I1* and *ALDH7B4* genes.

### Disruption of ALDH3I1 and ALDH7B4 affects Photosynthesis

**Figure 3:**
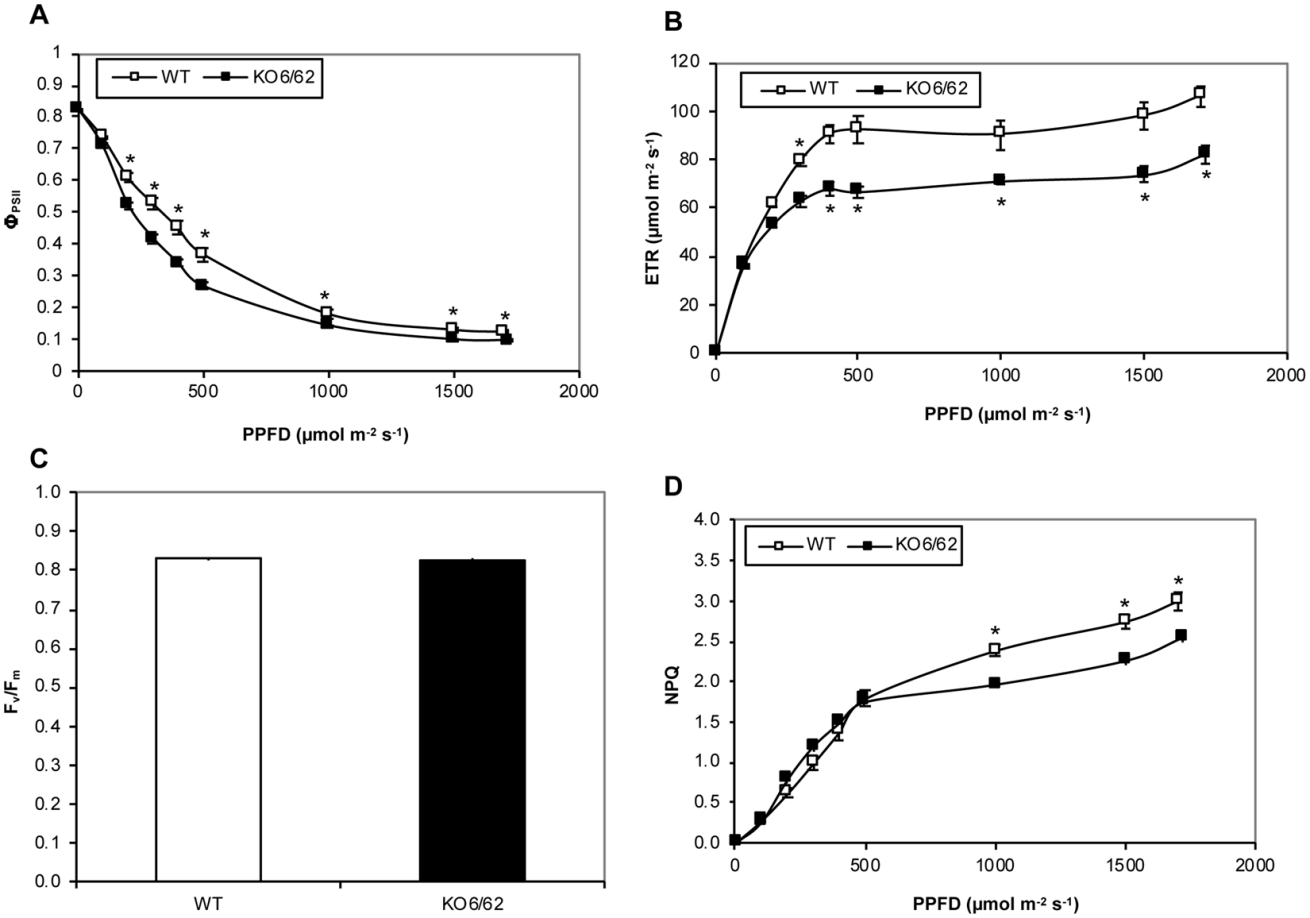
Chlorophyll fluorescence analysis in WT and the *aldh* mutant *KO6/62*. (**A**) Quantum yield of photosystem II (Φ_PSII_); (**B**) Electron transport rate (ETR); (**C**) Maximum quantum efficiency of the photosystem II (F_v_/F_m_); (**D**) Non-photochemical quenching (NPQ). Shown are mean values ± SE (n = 9 plants). Asterisks show significant differences according to Student’s *t* test (*P* < 0.05) between WT and the mutant *KO6/62*.

The low ratio of NAD(P)H/NAD(P) in the leaves of the double mutant of ALDHs led us to examine their photosynthetic capacity in comparison to that of WT. At light intensities above 200 µmol m^−2^ s^−1^, we found that the quantum yield of photosystem II (PSII) and the electron transport rate were low in *KO6/62* plants compared to WT (Fig. 3A,B), but there was no difference in the maximum quantum yield of PSII (F_v_/F_m_) of the two genotypes (Fig. 3C). The transport of electrons is coupled with that of protons and results in the formation of a trans-thylakoid proton gradient^25,26^. An increase in the proton gradient in turn downregulates the electron transport and upregulates the non-photochemical quenching (NPQ) mechanism of the photosystem II^27^. We found that the NPQ was similar between the WT and the mutant at light intensities less than 500 µmol m^−2^ s^−1^. At higher light intensities, the NPQ increased more slowly in the *KO6/62* mutant than in WT (Fig. 3D), suggesting a dysfunction in the non-photochemical mechanism, likely the xanthophyll cycle, in the ALDH double mutant.

## Discussion

ALDHs oxidize aliphatic and aromatic aldehydes to their corresponding carboxylic acids using NAD^+^ or NADP^+^ as cofactors^4^. Given that the oxidation of aldehydes by ALDHs generates reducing equivalents in the form of NADH or NADPH, we investigated how the ALDHs may influence the cellular redox homeostasis. For this purpose, we analyzed knockout mutants of ALDHs and found that the suppression of ALDHs resulted in the accumulation of the oxidized pyridine nucleotides and a decrease of the reduced forms. This was associated with a decrease in the NAD(P)H/NAD(P) ratio and an increase of the G6PDH activity in *KO6/62* adult leaves. The NAD(P)H/NAD(P) ratio and the G6PDH activity were however similar in the WT and *KO6/62* seedlings under control conditions (Fig. 1C,D), which indicates a contribution of ALDH-derived NAD(P)H to the homeostasis of pyridine nucleotides in the adult leaves of *A. thaliana*. In the plant cell cytosol, enzymes including the NADP-dependent Isocitrate Dehydrogenase (NADP-ICDH), Glucose 6-Phosphate Dehydrogenase (G6PDH), non-phosphorylating glyceraldehyde-3-phosphate dehydrogenase (GAPN), and the NADP-Malic Enzyme2 (NADP-ME2) may contribute to the NAD(P)H content. However, the NADP(H) pools were practically not affected in the Arabidopsis single mutants defective in either NADP-ICDH or NADP-ME2 enzymes^28,29^. With regards to this, the alteration of NAD(P)H in the *aldh* mutant thus indicates that the ALDHs show less redundancy with other enzymes producing NADPH for the cell redox homeostasis.

Pyridine nucleotides are important redox sensors, since they are associated with most reactive oxygen species (ROS)-detoxifying enzymes. ROS production has been demonstrated to be influenced by the NAD(P)H/NAD(P) ratio in the chloroplast or the mitochondria^30–33^. Indeed, the turnover of oxidized antioxidant pools such as glutathione and ascorbate depends on availability of NAD(P)H^23,24^. Our observation that the NADP(H)/NAD(P) ratio decreased in parallel with the reduced to oxidised glutathione ratio indicate that ALDHs would significantly contribute to the reduced glutathione cellular content by providing NADPH. It is noteworthy that both the total and the reduction state of glutathione decreased in the *aldh* mutant whereas a decrease in the reduction state of glutathione led to an increased total pool in the CATALASE2 mutant (*cat2*) defective in oxidant processing and *cat2gr1* double mutant defective in both oxidant processing and glutathione reduction by GLUTATHIONE REDUCTASE1 (GR1) in Arabidopsis^34^. Given that the level of lipid-peroxidation increased in the *aldh* mutants^4,7^, a rapid consumption of glutathione via Michael adduct formation with aldehydes would affect both the glutathione content and the cell redox status. Indeed, aldehydes including 4-hydroxynonenal (HNE) can be conjugated to glutathione via GLUTATHIONE S-TRANSFERASE (GST, EC 2.5.1.18) to form HNE-GST conjugates, and several GST isoforms were found to be induced in pumpkin (*Cucurbita maxima* Duch.) by aldehydes^35,36^. Thus, the decrease of total glutathione could well be due to both the altered NADPH and increased GST conjugation with the aldehydes that accumulate in the *aldh* mutant. Unfortunately, the amount of HNE-GST and aldehyde-GST conjugates in the *aldh* mutant were not quantifiable by the method of glutathione detection used in this study. This information will be very important to elucidate the fate of NAD(P)H generated by ALDHs. As shown for the cytosolic NADP-ICDH^28^, we speculate that this may serve as substrate either to GR1 for reducing oxidised glutathione or to the NADPH oxidase encoded by *AtRbohF* for the generation of oxidant under certain conditions. With regards to this, the limited increase of NAD(P)H/NAD(P) in the *KO6/62* mutant compared to WT when grown on agar plate supplemented with nitrate could imply that NAD(P)H-derived from the ALDH activity partly support nitrate assimilation. In fact, the NAD(P)H/NAD(P) ratio was similar in WT and *KO6/62* under control conditions and on medium supplemented with ammonium but not on medium supplemented with nitrate (Fig. 1). Although the increase of G6PDH activity in the *KO6/62* seedlings could compensate for the reduced NAD(P)H supply in the mutant, this was not sufficient, which suggests that the ALDH activities may have a pivotal role, besides photosynthesis, in contributing to the reducing power required for the nitrate assimilation. We found that the efficiency of photosynthesis decreased in the *aldh* mutant *KO6/KO62* compared to WT, which could also explain the diminution of NAD(P)H. It is however unclear how the mutation of the two ALDH genes might alter photosynthesis under control conditions. The reduced quantum yield of PSII and the increase in NPQ of the *aldh* mutant *KO6/62* in the absence of photoinhibition (Fig. 3D) at high-light conditions suggests a dysfunction of the xanthophyll cycle, which most likely result from the altered NAD(P)H contents in the mutant plants. While an analysis of photosynthetic pigments is required to confirm this thesis, it is noteworthy that the NAD levels were higher in the *aldh* mutants than in WT (Table 1). Such an increase of NAD was also observed in Arabidopsis *nadk2* mutant deficient in the chloroplast NAD kinase (EC 2.7.1.23) that phosphorylates NAD^+^ to NADP^+ ^^37–39^, and in which a low NAD(P)H/NAD(P) ratio was demonstrated to cause the inhibition of zeaxanthin epoxidase (EC 1.14.13.90) and the accumulation of zeaxanthin. A high level of zeaxanthin decreases the quantum yield of energy transfer from the antenna complex to the PSII reaction center, lowering thereby the photosynthetic yield. Previous reports also showed that both intrathylakoid pH and NADPH control the epoxidation reaction in the xanthophyll cycle^40,41^. The altered NAD(P)H/NAD(P) following an increase of NAD^+^ and a decrease of NAD(P)H in the *aldh* mutant has likely modified the redox state of the photosynthetic pigments, thereby affecting photosynthesis efficiency as shown in Fig. 3. Concurrently, several enzymes of the “dark reactions” of photosynthesis can be inactivated by carbonylation with the aldehydes that accumulate in the *aldh* mutant^4,7,42^. For instance, the Calvin cycle enzymes phosphoribulokinase, glyceraldehyde-3-phosphate dehydrogenase, fructose-1,6-bisphophatase, sedoheptulose-1,7-bisphosphatase, aldolase, and Rubisco were irreversibly inactivated by acrolein. Acrolein treatment caused a rapid drop of the glutathione pool, prior to the inactivation of photosynthesis. GSH exogenously added to chloroplasts suppressed the acrolein-induced inactivation of photosynthesis, but ascorbic acid did not show such a protective effect^43,44^. Thus, as a result of the mutation of ALDHs, aldehydes can accumulate and inhibit photosynthesis by depleting GSH and inactivating multiple enzymes in the Calvin cycle.

In conclusion, we have showed that the mutation of the ALDH3I1 and ALDH7B4 altered the cellular contents of NAD(P)H, the total and reduction state of glutathione, and decreased the efficiency of photosynthesis in *A. thaliana*. This is in agreement with the interdependency between the pyridine nucleotides, cellular redox, and photosynthesis and indicates a role of ALDHs as major contributors to the cellular redox homeostasis in *A. thaliana*.

## Methods

### Plant Materials and Growth Conditions

*Arabidopsis thaliana* (ecotype Col-0) was used throughout this work. Wild-type (WT) and transgenic plants were grown on half-strength-Murashige and Skoog (MS) agar plates^45^ or soil under white light. The soil was a 3:1 mixture of potting soil (Rolfs Gärtnereinkauf, Siegburg, Germany) and vermiculite. The intensity of the light was about 150 μmol m^−2^ s^−1^ with 8/16 h day/night-cycle at 22 to 24 °C. Leaves of fourteen-day- and six-week-old plants were immediately frozen in liquid nitrogen and used for analyses. The harvest for all samples was four hours after the light was switched on, as this should reflect stable day metabolites.

### Isolation of Homozygous T-DNA Insertion Mutants

Single mutants *KO6* (SALK_143309) for ALDH7B4 (*At1g54100*) and *KO62* (nos. 16843)^46^ for ALDH3I1 (*At4g34240*) were previously described^7^. Similarly, the single mutant *KO69* (nos. 22184)^46^ for ALDH3H1 (*At1g44170*) was described and referred as *3h1-B* in Missihoun *et al*.^47^. The single mutant *KO76* (SALK_091250) for ALDH3F1 (*At4g36250*) carries a T-DNA insertion in the fourth exon. Double mutants *KO6/62*, *KO6/76*, *KO6/69*, and *KO69/76* were obtained by crossing the corresponding homozygous single mutants. The second seed generation derived from the crossing was screened to isolate homozygous double mutants. Homozygous T-DNA insertion mutants were identified by two consecutive PCR assays. The first PCR assay involved the use of two gene-specific primers. Gene-specific primers comprised 5′-TTGCCAAGGGTTTTTCTCCTGCCAG-3′ and 5′-TAAGATCCGCGTCCCCTGAAAAGCT-3′ for *ALDH3I1*; 5′-CATACGAGGATGATCGTGGCAATG-3′ and 5′-TCACCTCTCTTAGGAGCCGTAACCT-3′ for *ALDH7B4*; 5′-ATCGGCGGAAGCGAGTAATTTGGTG-3′ and 5′-TATGGCGGATACCTGACGGCTGAATC-3′ for *ALDH3H1*; and 5′-GAAGCCATGGAAGCTATGAAGGAGAC-3′ and 5′-GTCTCTGTCTCTCACTTTCCCCCTT-3′ for *ALDH3F1*. In the second assay, one gene-specific primer (underlined above for each gene) was combined with one T-DNA specific primer: FISH1 (5′-CTGGGAATGGCGAAATCAAGGCATC-3′)^46^ for *ALDH3I1* and *ALDH3H1* or LBa3 (5′-ACCCAACTTAATCGCCTTGCAGCAC-3′)^48^ for *ALDH7B4* and *ALDH3F1*.

### RNA and Protein Analyses

Isolation of total RNA and RT-PCR analyses were performed as described before^3^. Gene-specific primers were used to amplify first strand cDNAs. These were 5′-GAAGCAATAGCCAAAGACACACGC-3′ and 5′-GATATCTCGATTATCGTAGGCTCC-3′′ for *ALDH7B4*, 5′-CTACTGGATGTGCCTGAAGCATC-3′ and 5′-TAAGATCCGCGTCCCCTGAAAAGCT-3′ for *ALDH3I1*, 5′-CAGCTAAAGAACTGGATGGCTC-3′ and 5′-TCAACCAACTAAGTCCATGTTTGA-3′ for *ALDH3H1*, and 5′-GAAGCCATGGAAGCTATGAAGGAGAC-3′ and 5′-GTCTCTGTCTCTCACTTTCCCCCTT-3′ for *ALDH3F1*. Transcripts of the Arabidopsis *ACTIN-2* (*At3g18780*) gene were used as reference^49^ and were amplified with the primers 5′-GGAATCCACGAGACAACCTATAAC-3′ and 5′-AGGAATCGTTCACAGAAAATGTTTC-3′.

Protein expression was examined by immunoblot detection. Crude protein extracts were separated on 12% SDS–polyacrylamide gels and electroblotted from the gel onto a nitrocellulose Protran BA-85 membrane (Whatman, Dassel, Germany) at 100 Volts for 1 h in pre-chilled protein-blot transfer buffer (25 mM Tris, 192 mM glycine, 20% [v/v] methanol)^50^. The membrane was stained with Ponceau red solution (0.2% [w/v] Ponceau S in 3% [w/v] trichloroacetic acid). Then, unspecific binding sites were blocked by incubating the membrane for 1 h at room temperature or overnight at 4 °C in blocking solution containing 0.1% (v/v) Tween-20 and 4% (w/v) non-fat dry milk powder dissolved in 1X TBS (20 mM Tris-HCl; 150 mM NaCl; pH 7.5). The membrane was probed with 5000-fold diluted ALDH7B4 antiserum^7^. The immunodetection assay was performed by using the ECL Plus Western Blotting detection Kit (Amersham, Braunschweig, Germany). Signals were detected under a CCD camera (Intelligent Dark Box II, Fujifilm Corporation, Tokyo, Japan).

### Chlorophyll Fluorescence and Gas Exchange Measurements

Measurements of chlorophyll fluorescence were performed using GFS-3000 Portable Gas Exchange Fluorescence System (LED-Array/PAM-Fluorometer 3055FL, Heinz Walz, Effeltrich, Germany). Parameters were set as follows: Photosynthesis Photon Flux Density (PPFD), 100 to 1800 µmol m^−2^ s^−1^; chamber temperature, 25 °C; flow rate, 750 µmol s^−1^; relative humidity, 60–70%; 350 µl L^−1^ CO_2_; 21% oxygen. Plants were allowed to adapt to the respective conditions for 15 minutes before starting measurement.

### Pyridine Nucleotides, Glutathione, and Enzyme Activity Measurements

Chemicals were obtained from Sigma-Aldrich. Approximately 50 to 100 mg fresh leaves was used for each assay, if not otherwise stated. Determination of NAD^+^, NADP^+^, NADH, and NADPH contents followed a previously described enzymatic cycling procedure^51,52^. Tissues were homogenized with 1 mL of 0.1 N KOH containing 50% ethanol (v/v) (for determination of NADPH and NADH) or 1 mL of 0.1 N HCl containing 50% (v/v) ethanol (for determination of NADP^+^ and NAD^+^). The suspension was heated for 5 min at 95 °C, cooled on ice and centrifuged (15,000 g) for 20 min at 4 °C. The oxidized and reduced forms of the nucleotides were determined from the supernatant or a standard solution corresponding to 0 to 500 pmol of NADP(H) in 1 mL reaction mixture containing 100 mM HEPES-KOH (pH 8.0), 0.5 mM EDTA, 2.5 mM glucose-6-phosphate (G6P), 1.66 mM phenazine ethosulfate, 0.42 mM 3-(4,5-dimethyl-2-thiazolyl)−2,5-diphenyl-2H-tetrazolium bromide. The reaction was started by adding 0.7 unit of glucose-6-phosphate dehydrogenase (G6PDH) enzyme. For the determination of NAD^+^ and NADH, the reaction mixture was the same except that Glc-6-P was replaced by 100 µl absolute ethanol. For the determination of NADH, the mixture contained 0.2 mM phenazine ethosulfate. The reaction was started by the addition of 0.7 unit of alcohol dehydrogenase. The rate of thiazolyl blue reduction was recorded by measuring the absorbance change at 570 nm. The pyridine nucleotides were measured in a GENESYS 10 UV spectrophotometer (Thermo Fisher Scientific, Waltham, MA, USA). The concentrations of each pyridine nucleotides in the extract were calculated from the corresponding standard curves obtained for pyridine nucleotide solutions per tissue fresh weight. Freshly extracted samples and standards were assayed at the same time. Glutathione measurement was performed in a Biotek Powerwave XS2 Spectrophotometer by following the procedure of Griffith^53^.

Total G6PDH activities were determined essentially as reported earlier^54,55^. Tissues were extracted in 100 mM Tris-HCl pH 8.0, 10 mM MgCl_2_, 5 mM EDTA pH 8.0, 1 mM phenylmethylsulphonyl fluoride, 10% glycerol, 15 µM NADP^+^. The assay reaction contains 50 mM Tris-HCl pH 8.0, 10 mM MgCl_2_, 150 µM NADP^+^, 3 mM Glc-6-P, and 10 to 20 µl extract. The reduction of NADP^+^ was measured through the increase of the absorbance at 340 nm. The activity was expressed as nmol NADPH min^−1^ mg^−1^ protein.

### Data availability

All data generated or analysed during this study are included in this published article (and its Supplementary Information file).

## Electronic supplementary material

Supplementary Information
