# Supplementary material for: Aldehyde Dehydrogenases Function in the Homeostasis of Pyridine Nucleotides in Arabidopsis thaliana
Source: Sci Rep. 2018 Feb 13;8:2936. doi: 10.1038/s41598-018-21202-6 (PMC5811564; doi:10.1038/s41598-018-21202-6)
Supplement: Supplementary file 1 — Supplementary Information [file 41598_2018_21202_MOESM1_ESM.pdf]

Title:

Aldehyde Dehydrogenases Function in the Homeostasis of Pyridine Nucleotides in *Arabidopsis thaliana*

Authors:

Tagnon D. Missihoun[1,4*], Simeon O. Kotchoni[2,3], Dorothea Bartels[1]

[1]Institute of Molecular Physiology and Biotechnology of Plants (IMBIO), University of Bonn, 53115 Bonn, Germany. [2]Department of Biology, Rutgers University, 315 Penn St., Camden, NJ 08102, USA. [3]Center for Computational and Integrative Biology, 315 Penn St., Camden, NJ 08102, USA.

[4]Present address: Department of Microbiology and Plant Pathology, University of California Riverside, Riverside CA 92521, USA.

[*]**Corresponding Author:** tagnon1980@gmail.com

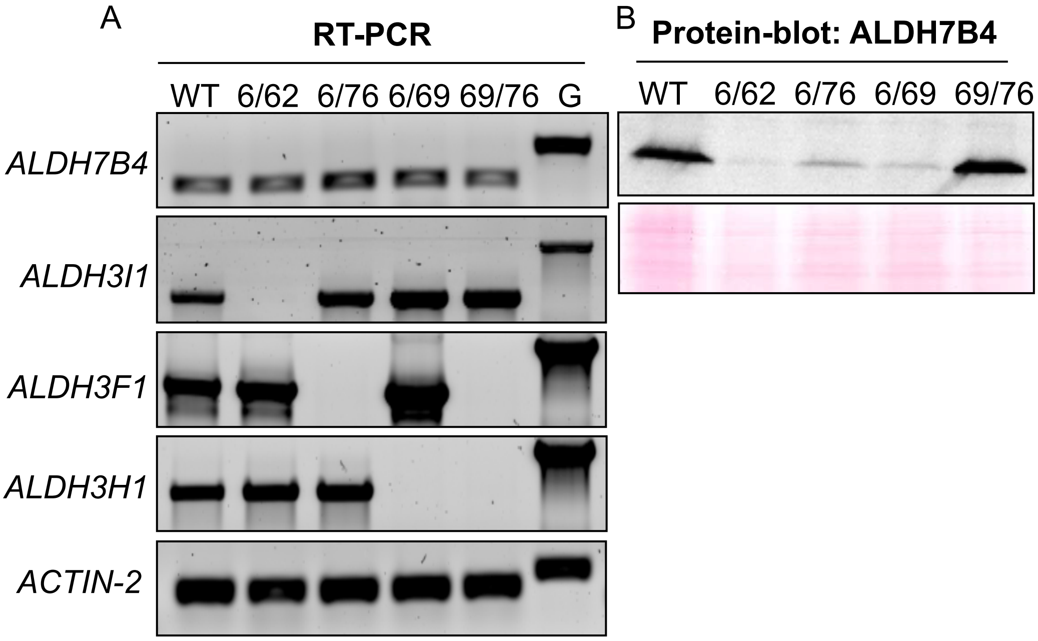

Suppl. Figure 1. Molecular analysis of the *ALDH* T-DNA insertion mutants. A, ALDH-specific transcript accumulation in the wild type Col-0 (WT) and *ALDH* double mutants: 6/62 for *ALDH7B4* and *ALDH3I1*, 6/76 for *ALDH7B4* and *ALDH3F1*, 6/69 for *ALDH7B4* and *ALDH3H1*, 69/76 for *ALDH3H1* and *ALDH3F1*. The *ACTIN-2* gene was used as a housekeeping gene control and G denotes genomic DNA from wild type Col-0 used as control for the reverse transcription reaction. B, (upper panel) Immunodetection of the ALDH7B4 protein (54 kDa) in WT and mutant seed extracts; (lower panel) Ponceau red staining of the membrane showing comparative loading of proteins of all samples.
